# Opposing effects of butyrate and bile acids on apoptosis of human colon adenoma cells: differential activation of PKC and MAP kinases

**DOI:** 10.1038/sj.bjc.6600793

**Published:** 2003-03-04

**Authors:** L McMillan, S K Butcher, J Pongracz, J M Lord

**Affiliations:** 1 Department of Immunology, The Medical School, Birmingham University, Birmingham B15 2TT, UK; 2 Department of Anatomy, The Medical School, Birmingham University, Birmingham B15 2TT, UK

**Keywords:** colon adenoma cancer, bile acids, butyrate, apoptosis, PKC, MAP kinase

## Abstract

Butyrate, produced in the colon by fermentation of dietary fibre, induces apoptosis in colon adenoma and cancer cell lines, which may contribute to protection against colorectal cancer. However, butyrate is present in the colon along with other dietary factors, including unconjugated bile acids, which are tumour promoters. We have shown previously that the proapoptotic effects of butyrate on AA/C1 human adenoma cells were reduced in the presence of bile acids. To determine the cellular basis of this interaction, we examined the effects of butyrate and the secondary bile acid ursodeoxycholic acid (UDCA) on signalling pathways known to regulate apoptosis using AA/C1 cells. Butyrate activated PKC-*δ* and p38 MAP (mitogen-activated protein) kinase, whereas UDCA activated PKC-*α* and p42/44 MAP kinase. Butyrate treatment also resulted in the caspase-3-mediated proteolysis of PKC-*δ*. Butyrate-induced apoptosis was reduced by inhibitors of PKC-*δ* (Rottlerin), p38 MAP kinase (SB202190) and caspase 3 (DEVD-fmk), whereas the proliferative/survival effects of UDCA were blocked by inhibitors of PKC-*α* (Gö6976) and MEK 1 (PD98059). The effects of butyrate and bile acids are therefore mediated by the differential activation of signalling pathways that are known to regulate apoptosis.

A diet high in dietary fibre and low in saturated fats is associated with a reduced incidence of colorectal cancer (Sandler *et al*, 1993). The benefits of a high intake of dietary fibre have been attributed to their fermentation in the gut to short-chain fatty acids, including butyrate. Butyrate has been shown to increase apoptosis in both colon adenoma and cancer cell lines in a p53-independent way (Hague *et al*, 1993), an effect that is likely to contribute significantly to its protective effects. In contrast, a diet high in saturated fats is associated with an increased level of faecal secondary bile acids (Radley *et al*, 1993) and an increased risk of colorectal cancer (Reddy and Wynder, 1977). Secondary bile acids, deoxycholic acid (DCA), ursodeoxycholic acid (UDCA) and lithocholic acid, are produced by the actions of intestinal bacteria on primary bile acids. Unconjugated bile acids have been shown to be tumour promoting in rodent colonic epithelium (Narisawa *et al*, 1974; Mahmoud *et al*, 1999).

The majority of studies in the literature concerning the effects of butyrate and bile acids on colonic epithelial cell proliferation and apoptosis have considered these dietary factors individually. *In vivo* both are present in the colon and may influence each other's actions directly or indirectly. For example, butyrate is known to modify colonic pH and inhibit the colonic bacteria responsible for the production of bile acids (MacDonald *et al*, 1978). We have shown previously that human adenoma cells, AA/C1, treated with physiological levels of bile acids showed increased proliferation (McMillan *et al*, 2000). Butyrate induced apoptosis in AA/C1 cells (Hague *et al*, 1993; McMillan *et al*, 2000) and addition of bile acids to these cells reduced the level of apoptosis, although this effect could be overcome by increasing the level of butyrate (McMillan *et al*, 2000). Therefore, these two dietary factors interact at the cellular level and the protective effect of butyrate may relate both to its apoptosis-inducing effect *per se* and its ability to modify the antiapoptotic, proliferative effects of secondary bile acids.

The molecular basis for the proapoptotic effects of butyrate have not been fully worked out, although it does not involve induction of Fas expression or modulation of bcl-2 family protein expression (Bonnotte *et al*, 1998; Giardina *et al*, 1999). However, a study by Medina *et al* (1997) has shown that butyrate induced activation of caspase 3 in colorectal cancer cells. Caspase 3 is a member of a cysteine protease family that are involved specifically with the initiation and execution of the apoptotic programme (reviewed in Kumar, 1995). Caspase 3 is a downstream effector caspase that is activated by upstream caspases, predominantly caspases 8 and 9. Caspase 3 can be activated by several routes, these include ligation of cell surface receptors, such as Fas (Enari *et al*, 1996), which are associated with caspase 8; or by factors released from mitochondria, that is, cytochrome *c* (Liu *et al*, 1996), in response to cellular stress, which in turn activate caspase 9 (Liu *et al*, 1996). Caspase 3 can also be activated by factors, including cellular stress, which leads to activation of the p38 MAP (mitogen-activated protein) kinase pathway, independent of the mitochondria (Shimizu *et al*, 1999).

The tumour-promoting effects of bile acids have been ascribed predominantly to their potent activation of protein kinase C (PKC) (Huang *et al*, 1992). PKC is a family of 11 isoenzymes that are differentially regulated and play specific roles in the control of cell proliferation, differentiation and apoptosis (Clemens and Trayner, 1992; Deacon *et al*, 1997). With respect to apoptosis, PKC-*α* and -*β*ll appear to be antiapoptotic in most cells. PKC-*α* is inactivated by proapoptotic factors including ceramide (Lee *et al*, 1996) and is known to phosphorylate bcl-2, potentiating its antiapoptotic function in mitochondria (Ruvolo *et al*, 1998). PKC-*β*ll is a mitotic lamin kinase (Goss *et al*, 1994) involved in the regulation of proliferation. In contrast, PKC-*δ* has a proapoptotic function and can be activated proteolytically by caspase 3 (Ghayur *et al*, 1996). Furthermore, transfection of cells with the caspase-3-generated fragment of PKC-*δ* is sufficient to induce apoptosis, while the same fragment mutated to inactivate the kinase domain is ineffective (Ghayur *et al*, 1996). In this study, we have considered the interaction between bile acids and butyrate by investigating the signalling pathways activated to effect their pro- and antiapoptotic actions.

## MATERIALS AND METHODS

### Cell culture

The colorectal adenoma cell line AA/C1 was provided by Professor C Paraskeva (Manning *et al*, 1991) and was cultured as described previously (Williams *et al*, 1990). Prior to treatment with bile acids or butyrate, cells were trypsinised and seeded at 10^6^ cells per flask, in triplicate, in DMEM (Gibco-BRL, Paisley, Scotland), containing 20% FCS (Sera Laboratories International, Crawley, UK), 2 mM glutamine, 0.2 U ml^−1^ insulin, 1 *μ*g ml^−1^ hydrocortisone sodium succinate (Sigma, Poole, UK), 100 U ml^−1^ penicillin and 100 *μ*g ml^−1^ streptomycin. After 3 days, the medium was replaced with a medium containing 6 mM sodium butyrate or 10 *μ*M UDCA (Sigma, UK). UDCA was added to the medium from a 10 mM stock in DMSO and solvent controls were used throughout.

### Assessment of PKC isoenzyme activation

PKC resides in the cytosol in its inactive state and translocates to the membrane fraction upon activation. Following treatment with bile acid and butyrate, cells were harvested by scraping, lysed and cytosol (soluble) and membrane (particulate) fractions were isolated by centrifugation as previously described (Griffiths *et al*, 1996). Briefly cell lysates were spun at 100 000 × **g** for 45 min at 4°C, the supernatant (soluble fraction) was removed and the pellet (particulate fraction) was extracted in lysis buffer (50 mM Tris-HCl, pH 7.4, 5 m, MgCl_2_, 1 mM EGTA, 1 m EDTA, 100 *μ*g ml^−1^ leupeptin, 10 *μ*g ml^−1^ pepstatin and 1 mM PMSF) containing 0.5% Triton X-100. Both fractions were then taken up in SDS sample buffer and proteins were separated on 10% SDS–PAGE gels. Protein content of each sample was determined prior to electrophoresis and 20 *μ*g was loaded per lane. Proteins were then transferred to PVDF membrane (Immobilon P, Millipore, UK) and the PKC isoenzymes were detected by immunoblotting. Rabbit polyclonal antibodies to PKC-*α*, -*β* (l and ll), -*δ* (Santa Cruz Biotechnology, US), -*ɛ*, -*η* and -*ζ* (Transduction Laboratories, US) and HRP-conjugated anti-rabbit IgG antibodies (Amersham International plc, UK) were used and blots were developed using enhanced chemiluminescence (ECL, Amersham, UK). Equivalent loading was confirmed using an anti-*β* actin antibody (Sigma, UK).

### Measurement of p38 and p42/44 MAP kinase activation

Activation of p38 and p42/44 MAP kinases was determined by immunoblotting of whole cell extracts using antibodies that detect the phosphorylated, active form of these kinases (Upstate Biotechnology, US). Blots were developed using ECL. Equivalent loading was confirmed using antibodies that detect total p38 and p42/44 MAP kinase.

### Measurement of apoptosis

In studies to determine the effect of inhibitors of caspase 3, PKC isoenzymes and MAP kinases on the regulation of apoptosis by bile acids and butyrate, inhibitors were included in the culture medium with these agents and apoptosis was measured after 72 h. A volume of 0.1 *μ*M Gö6976 (Calbiochem, UK) and 20 *μ*M Rottlerin (Calbiochem, UK) were used to inhibit PKC-*α* and *β* and PKC-*δ*, respectively, and 10 *μ*M PD98059 and 1 *μ*M SB202190 (Calbiochem, UK) were used as inhibitors of MEK 1, which lies upstream of p42/44 MAP kinase and p38 MAP kinase, respectively. To confirm the involvement of caspase 3 in butyrate-induced apoptosis, the caspase 3 inhibitor Ac-DEVD-fmk (Calbiochem, UK) was used at 10 and 50 *μ*M. The concentrations of inhibitors used were based on their IC_50_ values and our previous experience with these agents (Pongracz *et al*, 1999; Cross *et al*, 2000). Apoptosis was assessed by measuring annexin V binding and exclusion of propidium iodide, in both attached and floating cells, using a commercial kit (Boehringer-Mannheim, Germany) and FACS analysis.

### Statistics

Data are presented as mean±standard deviation (s.d.) and means were compared by Student's *t*-test. A value of *P*<0.05 was taken to indicate a significant difference between the mean values.

## RESULTS

### Effect of sodium butyrate and UDCA on apoptosis of AA/C1 cells

**Figure 1:**
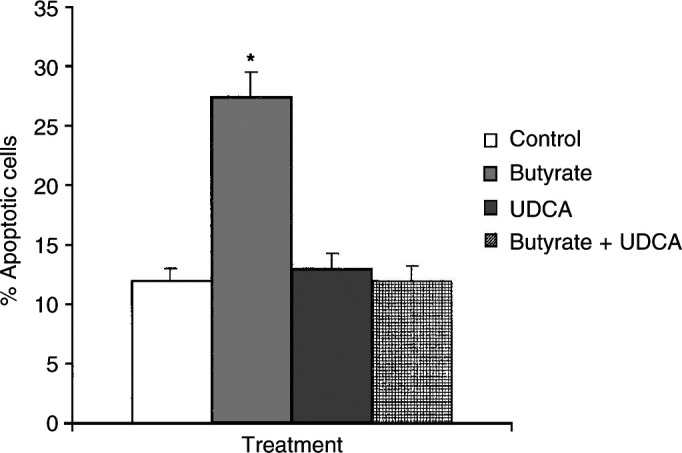
Effect of butyrate and UDCA on apoptosis in AA/C1 cells. Levels of apoptotic cells were determined in cultures of AA/C1 colon adenoma cells treated for 72 h with 6 mM sodium butyrate alone, 10 *μ*M sodium UDCA alone or butyrate and UDCA in combination. Apoptosis was assessed by binding of annexin V and exclusion of propidium iodide. Results are mean ±s.d. (*n*=3) and ^*^denotes *P*<0.005.

The basal level of apoptotic cells detected in AA/C1 cultures was 12.75±0.8%. As shown previously (Hague *et al*, 1993; McMillan *et al*, 2000), sodium butyrate (6 mM) induced a significant increase in apoptosis in AA/C1 adenoma cells (*P*<0.005; Figure 1

). In contrast, 10 *μ*M UDCA had no effect on spontaneous apoptosis of AA/C1, but did inhibit butyrate-induced apoptosis (Figure 1).

### Effect of sodium butyrate and UDCA on PKC isoenzyme activation

**Figure 2:**
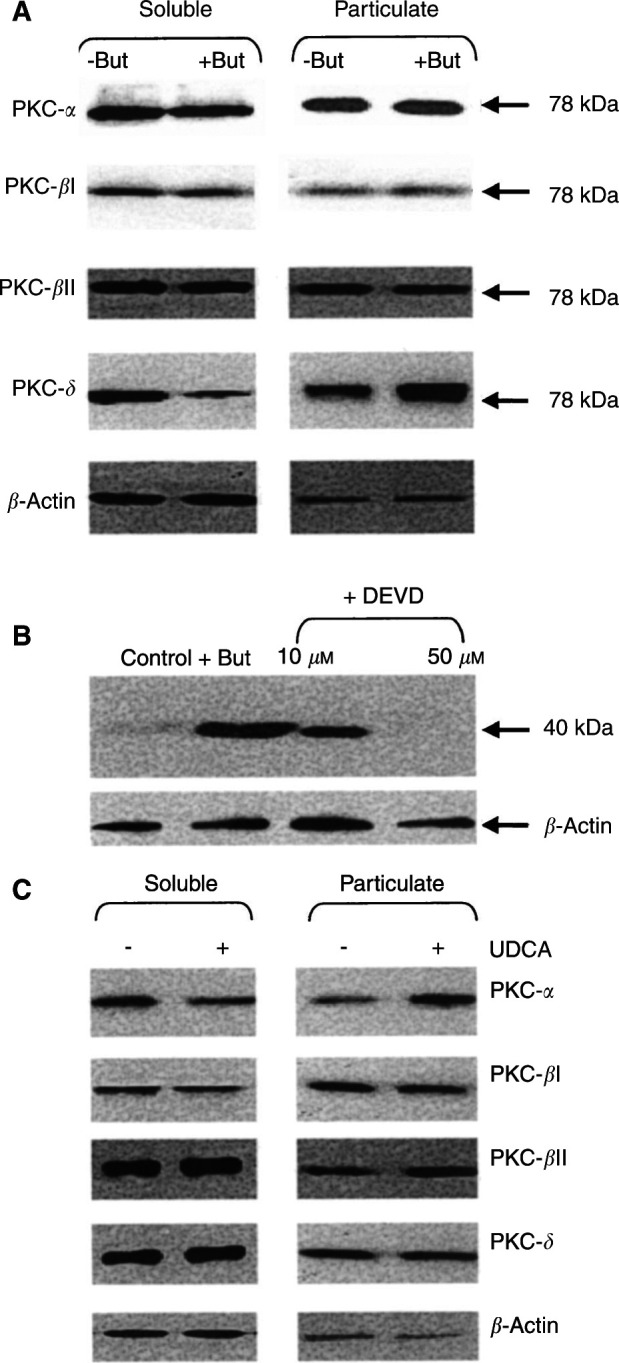
Effect of butyrate and UDCA treatment on PKC isoenzyme activation. PKC isoenzymes (*α*, *β*l, *β*ll and *δ*) were measured by Western blotting in soluble and particulate fractions of (**A**) AA/C1 cells treated with or without 6 mM sodium butyrate for 2 h or (**C**) 10 *μ*M UDCA for 24 h. (**B**) The 40 kDa fragment of PKC-*δ* was detected in whole cell lysates of AA/C1 cells treated for 18 h with 6 mM sodium butyrate in the absence or presence of the caspase 3 inhibitor Ac-DEVD-fmk. *β*-Actin was also measured as a loading control. The estimated molecular weights on the immunoreactive bands in (**A**) are shown on the right side of the figure. The blots shown are representative of three separate experiments performed.

PKC-*α*, *β*l, *β*ll and *δ* were detected in AA/C1 cells and were the predominant isoenzymes expressed in these cells (Figure 2A

). PKC-*ɛ*, -*η* and -*ζ* were also detected, but were expressed at a low level and were not affected by either bile acid or butyrate treatments (data not shown). Treatment of AA/C1 cells (Figure 2A) with 6 mM sodium butyrate induced a modest translocation of full-length 78 kDa PKC-*δ* from the soluble to the particulate fraction, 2 h after addition of butyrate. Densitometric analysis of blots from three separate experiments showed that the percentage of total PKC-*δ* present in the particulate fraction increased from 52.4±4% in untreated cells to 88.3±9% in butyrate-treated cells. Translocation of PKC-*α*, -*β*l and -*β*ll was not detected in response to butyrate treatment (Figure 2A), even up to 24 h after addition of butyrate (data not shown). Further studies revealed that a 40 kDa protein was detected by the anti-PKC-*δ* antibody in butyrate-treated cells. This fragment was not detected until 18–24 h of butyrate treatment and after the translocation of full-length PKC-*δ* (Figure 2B). PKC-*δ* can be proteolytically activated by caspase 3, leading to the generation of a constitutively active 40 kDa fragment (Ghayur *et al*, 1996). The appearance of the 40 kDa PKC-*δ* fragment was inhibited by inclusion of the caspase 3 inhibitor Ac-DEVD-fmk in butyrate-treated cultures (Figure 2B), suggesting that this represented the caspase activated form of PKC-*δ*.

Treatment of AA/C1 cells with 10 *μ*M UDCA induced translocation of PKC-*α* to the particulate fraction after 24 h, with no consistent change detected for the other PKC isoenzymes (Figure 2C). Densitometric analysis of three blots showed that the fraction of PKC-*α* associated with the particulate fraction increased from 27.4±4% in untreated to 59.8±7% in UDCA-treated cells. Thus, PKC isoenzymes were activated differentially by butyrate and UDCA.

### Effect of butyrate and UDCA on p38 and p42/44 MAP kinase activation

**Figure 3:**
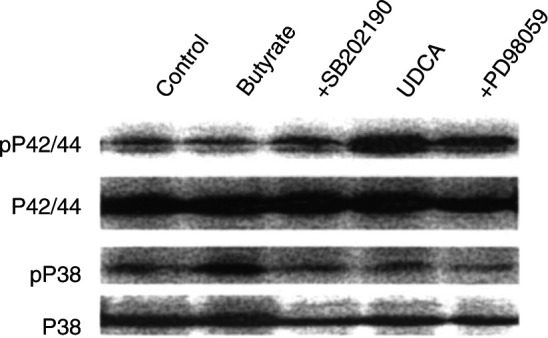
Effect of butyrate and UDCA on p38 and p42/44 MAP kinase activity. AA/C1 cells treated with 6 mM butyrate or 10 *μ*M UDCA for 24 h. Cells were examined for the presence of activated, phosphorylated p38 MAP kinase (pP38) and p42/44 MAP kinase (pP42/44) by Western blotting. PD98059 was also included with UDCA treatments (lane 5) to inhibit activation of p42/44 MAP kinase by MEK1, and SB202190 was included with butyrate treatments (lane 3) to inhibit p38 MAP kinase. Equal loading of gels was confirmed using an antibody to total P38 and P42/44 MAP kinase. The blot shown is a representative of three similar experiments performed.

Butyrate (6 mM) induced activation of p38 MAP kinase in AA/C1 cells, indicated by an increase in the level of phosphorylated p38 MAP kinase, but did not induce activation of p42/44 MAP kinase (Figure 3

). In contrast, active p42/44 MAP kinase was increased in cultures of AA/C1 treated with UDCA (Figure 3) and 10 *μ*M UDCA did not increase levels of activated p38 MAP kinase (Figure 3). Therefore, butyrate activated p38 MAP kinase, which has been shown to be involved in pathways leading to apoptosis and UDCA activated the p42/44 MAP kinase pathway, which has been implicated in cell proliferation and survival.

### Effect of PKC, MAP kinase and caspase 3 inhibitors on the actions of butyrate and UDCA

**Figure 4:**
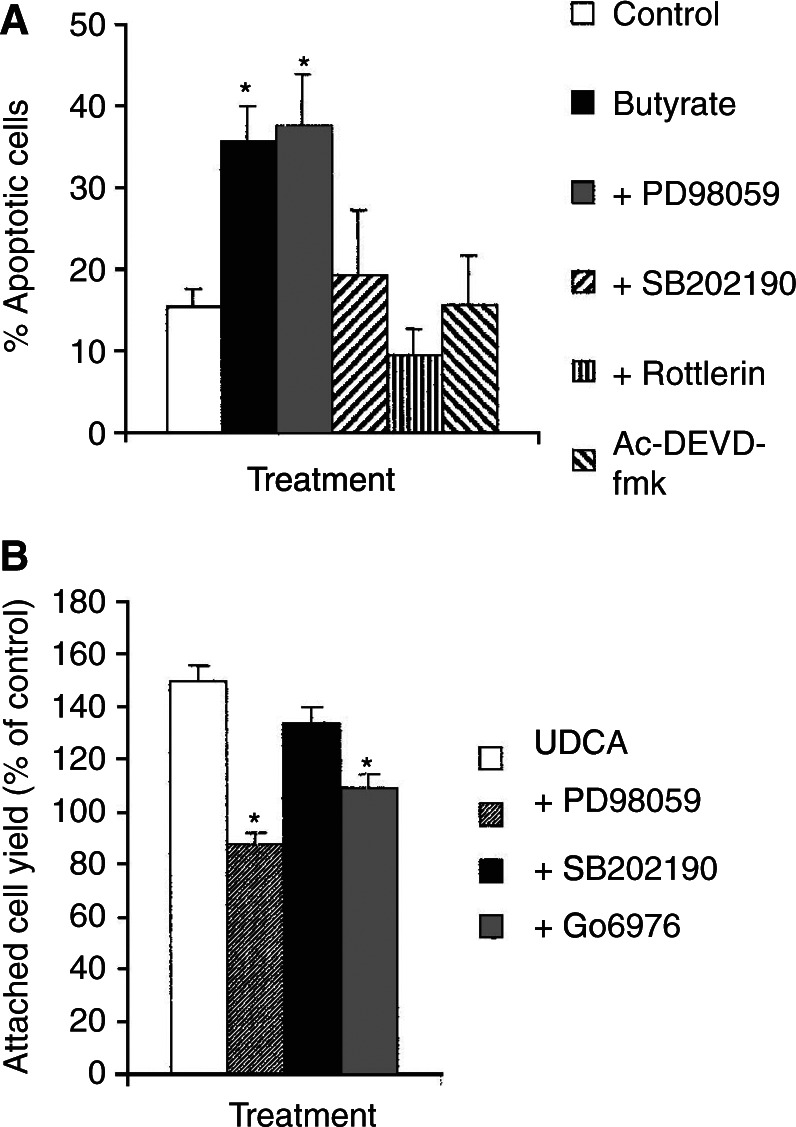
Effect of PKC, MAP kinase and caspase 3 inhibitors on the effects of UDCA and butyrate. (**A**) AA/C1 cells were incubated with 6 mM butyrate alone and in the presence of 10 *μ*M PD98059, 1 *μ*M SB202190, 20 *μ*M Rottlerin or 20 *μ*M Ac-DEVD-fmk. Apoptosis was measured after 72 h by annexin V binding and exclusion of propidium iodide. Values for butyrate treatment were compared with butyrate combined with the inhibitors used. (**B**) AA/C1 cells were incubated with 10 *μ*M UDCA in the absence or presence of 10 *μ*M PD98059, 1 *μ*M SB202190 or 0.1 *μ*M Go6976. Attached cell number was measured after 96 h and expressed as a percentage of the value for untreated cells. Data are mean ±s.d. of three separate experiments and ^*^denotes *P*<0.05 and ^**^denotes *P*<0.01.

To determine whether the signals through the PKC and MAP kinase pathways were required for the effects of butyrate and UDCA, inhibitors of these pathways were used. The PKC-*δ* inhibitor Rottlerin and the p38 MAP kinase inhibitor SB202190 blocked apoptosis induced by butyrate (Figure 4A

). As PKC-*δ* was also activated via caspase 3, the effects of the inhibitor Ac-DEVD-fmk were determined. Ac-DEVD-fmk also reduced apoptosis in butyrate-treated cultures (Figure 4A). The MEK 1 inhibitor PD98059 inhibits the activation of p42/44 MAP kinase indirectly and did not inhibit the proapoptotic actions of butyrate (Figure 4A). PD98059 blocked the effect of UDCA, inhibiting the increase in cell number seen with this bile acid (Figure 4B). The proliferative effect of UDCA was confirmed by measuring thymidine incorporation, which showed that incorporation of tritiated thymidine increased from 48.9±0.9 to 60.2±2.1 × 10^3^ d.p.m. per 10^5^ cells after 96 h treatment. The proliferative effects of UDCA were also reduced by Gö6976 (Figure 4B), an inhibitor of PKC-*α* and -*β* that does not affect PKC-*δ* (Gschwendt *et al*, 1996), confirming that both PKC and p42/44 MAP kinase are involved in mediating the effects of UDCA. The p38 MAP kinase inhibitor SB202190 had no effect on the proliferative effects of UDCA (Figure 4B). The inhibitor studies thus confirmed that modulation of PKC and MAP kinase signalling pathways by butyrate and UDCA was involved in mediating their effects on colorectal adenoma cell apoptosis and proliferation.

## DISCUSSION

In all tissues, including the colon, cell number and phenotype are maintained by a correct balance between cell proliferation, differentiation and apoptosis. Disruption of this balance underlies the development of cancer (Williams, 1991). In the colonic crypt, stem cells are located at the base and as they differentiate they progress up the crypt, eventually dying by apoptosis and are sloughed off into the colonic lumen (Potten and Grant, 1998). The high incidence of colorectal cancer in Western society has been attributed to a diet high in saturated fat and low in dietary fibre (Sandler *et al*, 1993). A high intake of saturated fat results in increased production of bile acids and raised levels of secondary bile acids, produced in the colon (Hill, 1986), have been measured in patients with adenomatous polyps and colorectal cancer (Radley *et al*, 1993). While bile acids are not mutagenic, they have been shown to act as tumour promoters in animal studies (Narisawa *et al*, 1974; Mahmoud *et al*, 1999) and PKC has been identified as their molecular target (Pongracz *et al*, 1995). We confirm here that the secondary bile acid UDCA was able to activate PKC in whole cells (Huang *et al*, 1992) and show that this action was restricted to the classical PKC isoenzyme PKC-*α*. Although UDCA was the focus of this study, we have also shown that other bile acids can inhibit butyrate-induced apoptosis, including CDCA (McMillan *et al*, 2000), and this bile acid also activates PKC-*α* (data not shown). Interestingly, the literature suggests that PKC-*α* has a predominantly antiapoptotic role (Deacon *et al*, 1997). For example, PKC-*α* is inhibited during ceramide-induced apoptosis (Lee *et al*, 1996) and expression of a dominant-negative PKC-*α* induced apoptosis in CHO cells (Whelan and Parker, 1998). Thus, those bile acids known to promote cell proliferation and inhibit butyrate-induced apoptosis, that is, UDCA, CDCA and DCA (Mahmoud *et al*, 1999; McMillan *et al*, 2000), are likely to be mediated by the activation of PKC-*α*. This proposal is supported by the ability of the PKC inhibitor Gö6976 to block the proliferative effects of UDCA on AA/C1 cells.

Our studies also revealed that a kinase lying downstream of PKC-*α*, namely p42/44 MAP kinase, was also activated following UDCA treatment. This kinase is the original member of the mitogen-activated protein kinase family (MAP kinases) that are activated by a variety of mitogens, including growth factors and the PKC activator TPA (Kyriakis *et al*, 1994). PKC-*α* is known to phosphorylate and activate Raf-1, a serine threonine kinase that activates the kinase upstream of p42/44 MAP kinase, MEK1 (Kolch *et al*, 1993). Inhibition of MEK1 was able to block the effects of UDCA, confirming that the PKC-*α* and p42/44 MAP kinase play a role in mediating the effects of bile acids on AA/C1 cells.

In contrast to the activation of survival/proliferative pathways by UDCA, butyrate was found to activate pathways known to mediate cell death by apoptosis, namely PKC-*δ* and p38 MAP kinase, consistent with the induction of apoptosis by this agent (Hague *et al*, 1993; McMillan *et al*, 2000). Butyrate has also been shown to activate p38 MAP kinase in Caco-2 cells (Ding *et al*, 2001). Loss of PKC-*δ* protein expression has been reported in human adenocarcinoma tissue (Craven and deRubertis, 1994) and this may underlie the reduced responsiveness of cancer tissue to the apoptosis-inducing effects of butyrate (Bonnotte *et al*, 1998). Butyrate has been shown previously to activate NF-*κ*B in a PKC-dependent manner, although the PKC isoenzyme involved was not determined (Giardina *et al*, 1999). A signalling pathway involving both p38 MAP kinase and NF-*κ*B and leading to activation of caspase 3 has been described (Ichijo *et al*, 1999; Shimizu *et al*, 1999). Our data suggest therefore that activation of full-length PKC-*δ*, which occurred before caspase 3 activation of PKC, may initiate the signals leading to p38 MAP kinase and NF-*κ*B activation. The latter will then result in caspase 3 activation and further activation of PKC-*δ* by caspase-3-mediated proteolysis. Although not investigated here, butyrate is also known to induce caspase 3 activation via the mitochondrial route. The latter involves generation of reactive oxygen species and release of cytochrome *c* from the mitochondria. Thus, caspase 3 activation by butyrate is achieved by two pathways and will ensure that cleavage and activation of the proapoptotic PKC-*δ* is achieved.

The targets of PKC-*δ* that effect its involvement in apoptosis have not been fully described, but appear to include predominantly nuclear proteins. The caspase-3-activated form of PKC-*δ* has been shown to inhibit DNA-PK, a nuclear protein involved in DNA repair (Bharti *et al*, 1998) and to phosphorylate nuclear lamin B prior to disassembly of the nuclear lamina (Cross *et al*, 2000). Although translocation of PKC-*δ* was shown here, the particular membrane involved was not identified. However, we have shown previously that PKC-*δ* translocated to the nuclear membrane during apoptosis in T cells, HL60 cells and neutrophils (Pongracz *et al*, 1999; Scheel-Toellner *et al*, 1999; Cross *et al*, 2000).

In summary, we have shown that the opposing effects of bile acid and butyrate, used at concentrations within the physiological range, on colon adenoma cell apoptosis are mediated via differential activation of signalling pathways that regulate apoptosis. The ultimate level of apoptosis in the colon may therefore be dictated by the balance of signals through the pro- and antiapoptotic PKC isoenzymes and MAP kinases. Such a proposal is supported by the data of Ding *et al* (2001), which showed that butyrate-induced apoptosis of Caco-2 cells was potentiated by the MEK inhibitor PD98059 and involved activation of p38 MAP kinase as reported here. Any beneficial effects of butyrate with regard to colon cancer will only be realised if levels of this agent are high enough in the colon to overcome the tumour-promoting signals induced by unconjugated bile acids.

Future studies will determine whether the signalling pathways activated by bile acids are able to inhibit directly the activation of proapoptotic signalling pathways, PKC-*δ* and p38 MAP kinase, activated by butyrate, or whether they act downstream of these signals, for example by inducing the phosphorylation and activation of bcl-2 (Ruvolo *et al*, 1998).
